# Influencing factors of public willingness to implement cardiopulmonary resuscitation: A mixed-methods systematic review

**DOI:** 10.1016/j.ijcrp.2025.200519

**Published:** 2025-09-24

**Authors:** Yajie Wang, Fangqiu Zheng, Jingju Xia, Limin Zheng, Dantong Wang, Haili Wang, Bo Ma

**Affiliations:** a People's Hospital of Liaoning Province, Liaoning, China; b School of Nursing, Liaoning University of Traditional Chinese Medicine, Liaoning, China

**Keywords:** Cardio-pulmonary resuscitation, Willingness, Influencing factors, Mixed methods, Systematic review

## Abstract

**Background:**

Bystander cardiopulmonary resuscitation is a key element in the chain of survival for out-of-hospital cardiac arrest, enhancing patient survival rates. However, bystander cardiopulmonary resuscitation rates remain low.

**Aim:**

(s)To systematically evaluate the factors influencing bystander cardiopulmonary resuscitation and provide a foundation for developing training programs and guiding future research.

**Methods:**

The systematic review adhered to the Preferred Reporting Items for Systematic Reviews and Meta--Analyses (PRISMA) guidelines and was registered with PROSPERO.

**Data sources:**

A comprehensive search of 4 databases (the Cochrane Library, PubMed, Web of Science, and Embase) were searched between the database's creation and November 13, 2024.

**Results:**

A total of 11,730 studies were initially retrieved, with 28 ultimately included in the analysis. Among these, 4 were qualitative studies, and 24 were quantitative studies. Data integration was performed using Nvivo14 software.

**Conclusion:**

The findings demonstrated that cardiopulmonary resuscitation (CPR) knowledge, abilities, training experience, rescue objects, and rescue techniques all had an impact on the public's willingness to perform CPR. Ethics teaching will be included in the training content, and future courses will use female simulators. Second, to address the issue of a lack of CPR knowledge, CPR instruction can be included in the curriculum of schools in nations such as China which have a poor rate of CPR training development. To increase the public's rate of mastery of CPR-related knowledge, the training frequency should be increased concurrently. And it is suggested that the state create relevant legislation to safeguard rescuers. Finally, designing specialized evaluation instruments.

## 1 Introduction

Out-of-hospital cardiac arrest (OHCA) is the sudden loss of systemic circulatory function caused by the loss of heart mechanical function outside of hospitals and other health care institutions. OHCA is one of the main causes of death worldwide because of its high incidence and low survival rates. There are over 275,000 OHCA cases annually in Europe [1], 24,500 OHCA cases annually in Spain, with death rates ranging from 79.9 % to 84.3 % [2], and approximately 700,000 OCHA cases annually in the U.S. [3]. The OHCA survival chain serves as the regional cooperative rescue system's practical manual for first aid situations. The American Heart Association developed the idea of the chain of survival in OCHA to highlight the optimal rescue strategy for out-of-hospital cardiac arrest, which includes early dial-120, high-quality CPR, early electrical defibrillation, early advanced life support, and thorough post resuscitation treatment [4]. According to worldwide resuscitation guidelines, bystander cardiopulmonary resuscitation (BCPR) is one of the crucial links in the chain of survival and is linked to a better prognosis following OHCA [5,6]. BCPR more than doubles the survival rate of cardiac arrest patients [7,8]. The implementation rate of BCPR is less than 50 % [9,10], with implementation rates of less than 5 % in Korea, 47.5 % in the U.S., and 10.5 %–40.9 % in Asian countries, despite the effectiveness of bystander rapid-initiated cardiopulmonary resuscitation [11,12]. The American Heart Association and the National AcadeMy of Medicine have stated that enhancing BCPR instruction and practice is a national priority [[13], [14], [15]]. Although bystander interventions, including BCPR, are being promoted and implemented extensively in many countries [6], barely half of OHCA patients obtain BCPR [16,17]. Therefore, it is crucial to determine the pertinent aspects influencing BCPR to further increase the number of patients undergoing BCPR and the effect that follows OHCA. Only researchers such as Tasuku [18] have examined the elements that influence bystanders who perform cardiopulmonary resuscitation outside of a hospital using the scope review technique In 2020, however, the study findings were subject to limitations, including the absence of a comprehensive systematic evaluation and rigorous bias assessment, which may affect the generalizability and reliability of the results. The mixed-methods systematic evaluation approach rigorously and scientifically identified the prominent factors influencing the implementation rate of out-of-hospital cardiopulmonary resuscitation. Therefore, to assess and identify important factors influencing public CPR performance, this study used a mixed-methods systematic evaluation approach. This will help guide future research activities and build future CPR training programs.

## 2 Aim

This study analyzed the factors influencing the rescue rate of out-of-hospital cardiopulmonary resuscitation (CPR) by a mixed methods systematic review to provide a basis for the subsequent development of CPR training courses or related research.

## 3 Methods

The protocol for this systematic review was prospectively registered with PROSPERO (International Prospective Register of Systematic Reviews; registration number CRD42025634676). The review process and reporting were conducted following the PRISMA (Preferred Reporting Items for Systematic Reviews and Meta-Analyses) statement guidelines [19].

### 3.1 Search strategy

The search mode in this study is determined by mixing subject terms with free words. The four databases of PubMed, Cochrane Library, WOS, and Embase were searched between the database's creation and November 13, 2024. Furthermore, reference tracing and an evaluation of pertinent systems were added. In Supplementary Material-1, the particular search mode is displayed.

### 3.2 Study selection

The inclusion criteria were as follows: age ≥18 years; the study's contents were connected factors influencing the public cardiopulmonary test rescue rate; the literature was in English; and the research techniques were mixed, quantitative, and qualitative.

The exclusion criteria were as follows: unable to extract the influential factors of the literature; studies from the same research team whose content was basically the same; unable to obtain the full text of the literature; duplicate publications; and case reports, policies and regulations, meeting minutes.

Independent researchers carried out all screening: four investigators (Yajie Wang, Fangqiu Zheng, Limin Zheng, and Dantong Wang) were in charge of full-text screening, whereas three researchers (Bo Ma, Jingju Xia, and Haili Wang) were in charge of titles and abstracts. The seven researchers debated their differences until they came to an agreement.

### 3.3 Quality assessment

The Critical Assessment Skills Program (CASP) qualitative checklist was used to evaluate the quality of the qualitative studies [20]. Regardless of study design, quantitative studies were evaluated using the Center for Evidence-based Stewardship's (CEBMa) critical assessment of survey instruments [21]. Both tools assist in the examination of internal and external validity and facilitate the assessment of methodological quality and transparency of reporting. Yajie Wang and Dantong Wang, two researchers, independently evaluated the quality of each study. In cases of disputed literature, resolution can be achieved through discussion or by the judgment of a third researcher (Bo Ma). Studies were classified as low quality if they fulfilled fewer than 65 % of the criteria, moderate quality if they met 65 %–90 %, and high quality if they met more than 90 %.

### 3.4 Data extraction

Two researchers (Yajie Wang and Dantong Wang) extracted the relevant information from the included literature, which was then verified by one other researcher (Haili Wang). The extracted data included the author, publication year, country, study type, and data collection methods.

### 3.5 Data analysis and synthesis

The data analysis employed the convergent integration approach of mixed methods systematic review [22]. Initially, quantitative data addressing the review questions were transformed into textual descriptions, which were then analyzed alongside qualitative data. This preliminary synthesis approach was utilized to explore the relationships between studies employing different methodologies [22]. The results sections of the qualitative studies, with the textual data from the quantitative and mixed-methods studies, were imported into NVivo 14. Codes were inductively generated, organized, and refined through an iterative process until sub-themes and themes emerged.

## 4 Results

**Fig. 1:**
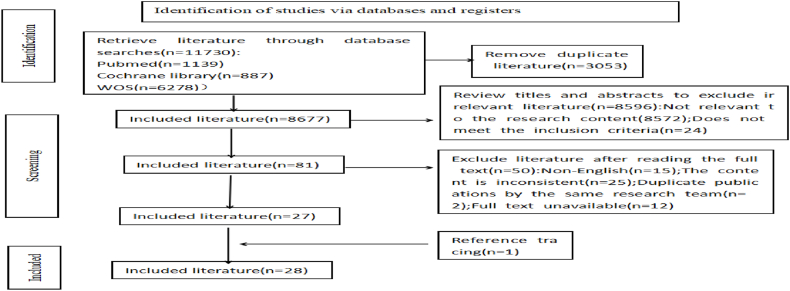
Flowchart of study selection.

The literature search process for this study is illustrated in Fig. 1. The initial search yielded 11,730 potentially relevant articles. Following the removal of duplicate records and subsequent screening of titles and abstracts, 81 articles were selected for further evaluation. After comprehensive full-text review, 27 articles met the inclusion criteria. Additionally, one relevant article was identified through reference tracking of the selected literature, resulting in the final inclusion of 28 articles for analysis.

### 4.1 Study characteristics

The final analysis included 28 studies, including 4 qualitative investigations and 24 quantitative analyses. The fundamental characteristics of these selected studies are summarized in Table 1.

**Table 1:** Fundamental characteristics of the included studies.

Authors and years	Country	Study Type	Data Collection Methods	Instruments	Reliability and Validity
Charlton, K.et al., 2023 [23]	England	Qualitative Research	Semi-structured Interview	Interview outline	–
Lu, C.et al., 2016 [24]	China	Quantitative Research	Questionnaire Survey	Self-made questionnaire	Test-retest reliability = 0.730
Son, J. W.et al., 2017 [25]	Korea	Qualitative Research	Structured Interview	Interview outline	–
Axelsson, A.et al., 2000 [26]	Sweden	Quantitative Research	Questionnaire Survey	Self-made questionnaire	–
Dobbie, F.et al., 2018 [27]	England	Qualitative Research	Face-to-Face Interview	Interview	–
Hung, M. S. Y.et al., 2017 [28]	China	Quantitative Research	Questionnaire Survey	Self-made questionnaire	the content validity index (CVI) = 0.945
Shams, A.et al., 2016 [29]	Lebanon	Quantitative Research	Questionnaire Survey	Self-made questionnaire	–
Swor, R.et al., 2006 [30]	America	Qualitative Research	Semi-structured Interview	Interview outline	–
Urban, J.et al., 2013 [31]	Korea	Quantitative Research	Questionnaire Survey	Self-made questionnaire	–
Al-Riyami, H.et al., 2020 [32]	Oman	Quantitative Research	Questionnaire Survey	Self-made questionnaire	–
Lu, C.et al., 2017 [33]	China	Quantitative Research	Questionnaire Survey	Self-made questionnaire	Test-retest reliability = 0.73
Chen, Y.et al., 2024 [34]	China	Quantitative Research	Questionnaire Survey	Self-made questionnaire	Cronbach's α = 0.814
Johnston, T. C.et al., 2003 [35]	Australia	Quantitative Research	Questionnaire Survey	Self-made questionnaire	–
Riccò, M.et al., 2020 [36]	Italy	Quantitative Research	Questionnaire Survey	Self-made questionnaire	–
Lee, M. J.et al., 2013 [37]	Korea	Quantitative Research	Questionnaire Survey	Self-made questionnaire	–
Alwidyan, M. T.et al., 2023 [38]	Jordan	Quantitative Research	Questionnaire Survey	Self-made questionnaire	–
Karuthan, S. R.et al., 2019 [39]	Malaysia	Quantitative Research	Questionnaire Survey	Self-made questionnaire	–
Chew, K. S.et al., 2019 [40]	Malaysia	Quantitative Research	Questionnaire Survey	Self-made questionnaire	Cronbach's α = 0.930,correlation coefficient = 0.930
Mao, J.et al., 2021 [41]	China	Quantitative Research	Questionnaire Survey	Self-made questionnaire	Cronbach's α = 0.820
Coons, S. J.et al., 2009 [42]	America	Quantitative Research	Questionnaire Survey	Self-made questionnaire	–
Charlton, K.et al., 2022 [43]	England	Quantitative Research	Questionnaire Survey	Self-made questionnaire	–
Bray, J. E.et al., 2017 [44]	Australia	Quantitative Research	Questionnaire Survey	Self-made questionnaire	–
Anto-Ocrah, M.et al., 2020 [45]	Ghana	Quantitative Research	Questionnaire Survey	Self-made questionnaire	–
Chen, M.et al., 2017 [46]	China	Quantitative Research	Questionnaire Survey	Self-made questionnaire	–
Pei-Chuan, H. E.et al., 2019[^47]^	China	Quantitative Research	Questionnaire Survey	Self-made questionnaire	kappa coefficient values for two items were between 0.4 and 0.6 and greater than 0.6 for the remaining items, content validity>90 %
Kuramoto, N.et al., 2008 [47]	Japan	Quantitative Research	Questionnaire Survey	Self-made questionnaire	–
Gul, S.et al., 2019 [48]	America	Quantitative Research	Questionnaire Survey	Self-made questionnaire	–
Sipsma, K.et al., 2011 [49]	America	Quantitative Research	Questionnaire Survey	Self-made questionnaire	–

"-"not mentioned.

### 4.2 Quality assessment

The quality assessment of the included studies is detailed in Supplementary Material-2. The quality evaluation revealed that the overall methodological quality ranged from low to moderate, with only one study demonstrating high quality [23]. Among the four qualitative studies, all presented clear statements, appropriate research designs, and proper qualitative methodologies; however, none addressed the relationship between researchers and participants. Among the 24 quantitative studies, while all articulated clear research questions and employed appropriate study designs, a substantial proportion (46 %, 11/24) were of low methodological quality.

### 4.3 The research methods make the research results heterogeneous

Self-designed questionnaires were used in all 28 included studies. Only 7 of these studies examined validity and reliability, and none tested sensitivity or specificity.

### 4.4 Facilitators of public willingness to perform out-of-hospital cardiopulmonary resuscitation

**Fig. 2:**
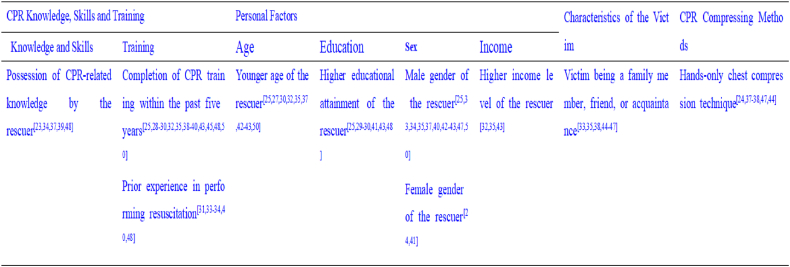
Facilitators influencing public willingness to perform cardiopulmonary resuscitation.

**Fig. 3:**
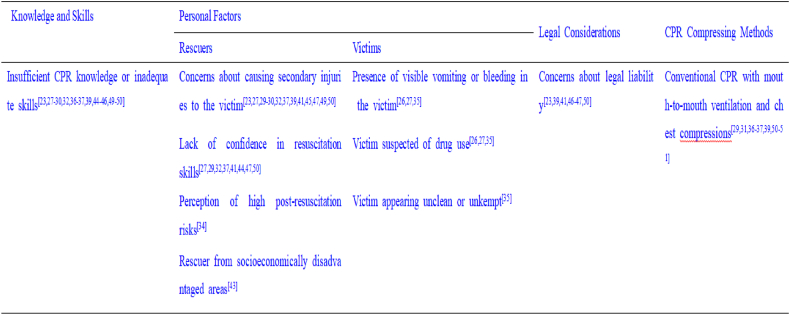
Barriers influencing public willingness to perform cardiopulmonary resuscitation.

Charlton et al. [23] employed semistructured interviews and reported that individuals with CPR knowledge demonstrated greater willingness to assist cardiac arrest victims, a finding that is consistent with research conducted in Malaysia [39]. Their study identified CPR training [28,29,31,32,[37], [38], [39], [40],^42^,^44^,^50^] and prior rescue experience [33,34,40,47] as primary facilitators, with more frequent educational exposure and CPR training within the last five years correlating with greater willingness to perform resuscitation [25,30,35,37,49]. The willingness to perform CPR varies depending on the victim's relationship with the rescuer, with greater willingness observed when the victim is a family member, friend, or acquaintance, prompting rescuers to initiate CPR before emergency medical services arrive [24,35,37,38,[44], [45], [46], [51],^52^,^53^]. With respect to personal factors, gender, age, educational level, and income significantly influence CPR willingness. Both Lu, C [24] and Mao, J.'s [41] studies indicate that women are generally more willing to perform CPR during cardiac arrest than men are, although some studies suggest that men are more likely to perform CPR in public settings [25,33,37,42]. Younger age and higher education levels are positively associated with increased willingness to perform CPR [25,27,30,32,33,37,47]. A Lebanese study [29] further revealed that individuals with higher income levels demonstrate greater willingness to assist cardiac arrest victims. A literature review revealed that, compared with traditional CPR procedures, the public shows greater preference for hand-only chest compression as a pre-hospital resuscitation method for cardiac arrest patients[^24,31,38,41,42,47,53]^. The detailed findings are presented in Fig. 2, Fig. 3.

### 4.5 Barriers to public engagement in out-of-hospital cardiopulmonary resuscitation

This study revealed that the primary barriers to CPR implementation are a lack of CPR knowledge and unfamiliarity with resuscitation procedures, which significantly deter public engagement in life-saving interventions [23,[27], [28], [29], [30],^32^,^36^,^37^,^39^,^41^,^46^,^48^,^49^,^52^]. To address these barriers, it is imperative to develop region-specific, targeted training programs that increase public CPR proficiency, thereby potentially increasing the rate of bystander CPR administration. Another substantial barrier is concern about causing secondary injuries to victims during resuscitation attempts [23,27,29,30,32,37,39,41,45,48,49,52]. Furthermore, studies have revealed that potential legal consequences represent a significant deterrent [23,29,36,39,41,46,51,49,[52], [53], [54]], as bystanders may hesitate to perform CPR due to fears of subsequent litigation. While there is no legal obligation for bystanders to provide assistance, rescuers may face legal action if CPR is performed improperly.

## 5 Discussion

This study employed a mixed-methods systematic review approach to identify and synthesize the facilitators and barriers influencing public engagement in out-of-hospital cardiopulmonary resuscitation. The analysis revealed that the key determinants encompass five primary dimensions: CPR knowledge, skills, and training experience; characteristics of the victim; methods of CPR; individual factors; and legal considerations. Additionally, all the research used self-made questionnaires for their surveys, which resulted in a lack of consistency and significant data variability.

### 5.1 The impact of CPR knowledge, skills, and training experience on resuscitation willingness

OHCA presents a critical public health challenge, characterized by low survival rates and poor prognosis, and poses a significant threat to population health [54]. Prompt and effective CPR serves as the primary intervention for OHCA patients, with early CPR administration playing a pivotal role in determining patient outcomes [8]. However, research indicates that fewer than one-third of OHCA victims receive BCPR [8], reflecting suboptimal rates of pre-hospital resuscitation. This mixed-methods systematic review analyzed 28 studies investigating factors influencing CPR willingness and revealed that a lack of CPR knowledge and skills constitutes a significant barrier to bystander intervention [23,27,29,30,32,36,37,45,46,48,52]. When encountering cardiac arrest situations, individuals lacking CPR proficiency often experience fear, anxiety, and hesitation, inhibiting their willingness to perform resuscitation [55]. These findings align with previous research [27,29] emphasizing the correlation between CPR knowledge deficits and a reduced willingness to intervene. CPR knowledge and skills function as dual determinants, serving as both facilitators of and barriers to willingness to resuscitate. Public acquisition of CPR competencies enhances confidence and increases the likelihood of intervention. CPR training has emerged as the primary modality for public education Hamasu et al. [56] demonstrated that trained individuals possess greater knowledge and are more likely to administer CPR during cardiac arrest events. Zhou et al. [57] further corroborated that adequate CPR knowledge and skills significantly influence the willingness to intervene. The temporal aspect of training retention warrants attention, as studies indicate that knowledge decays within 6–12 months post training, corresponding with reduced bystander willingness [[58], [59], [60]]. Sipsma et al. [49] observed significantly increased CPR willingness following three or more training sessions. Similarly, qualitative research by Study Son et al. [25] reported that participants who received two or more educational interventions demonstrated markedly increased willingness to perform CPR, with those who underwent four or more CPR education sessions exhibiting sevenfold greater willingness than untrained individuals did. These findings underscore the importance of implementing recurrent training sessions and maintaining regular refresher courses to increase both the training rate and the likelihood of CPR administration during cardiac arrest events. This study recommends that nations with underdeveloped CPR training programs, particularly China, implement government-mandated CPR training within their secondary and tertiary education systems to enhance bystander resuscitation capabilities. Furthermore, educational-based language issues are often obscure and difficult for the general public to understand. Future CPR training programs and curriculum development should prioritize linguistic accessibility to facilitate public understanding and engagement.

### 5.2 The impact of victim characteristics on bystanders' willingness to perform resuscitation

Bystanders' willingness to perform CPR significantly varies depending on the victim's characteristics. The attitudes and actions of family members, friends, and coworkers regarding helpful behaviors can also serve as catalysts, motivating others to participate in such behaviors [61]. When cardiac arrest occurs in family members, friends, or acquaintances, bystanders demonstrate a greater propensity to intervene actively and administer CPR [61], demonstrate lower CPR rates for strangers [23,38,[45], [46], [51],^52^,^57^] or are uncertain about performing CPR on strangers, with half citing health and safety concerns [62]. Furthermore, bystanders exhibit reluctance when victims present with vomiting, visible blood, an unclean appearance, or signs of alcohol consumption. Previous research has shown that victim characteristics, such as vomiting around the mouth or bloodstains, constitute significant barriers to CPR provision [[63], [64], [65]]. The literature review also identified gender-specific reluctance, particularly with respect to female victims, primarily due to concerns about compromising personal privacy. Kramer et al.'s [66] simulation study revealed laypersons' hesitation to remove clothing from female manikins during CPR training. These findings suggest several practical implications for CPR training programs: (1) incorporation of female manikins to address privacy concerns,(2) development of national policies to mitigate potential complaints or legal issues,(3) educational components addressing common cardiac arrest symptoms (e.g., vomiting, fainting) to reduce public apprehension; and (4) integration of ethical training modules to enhance moral reasoning and promote altruistic behaviors toward strangers.

### 5.3 The impact of resuscitation techniques on bystanders' willingness to intervene

Qualitative research [23] findings indicate that mouth-to-mouth resuscitation constitutes a significant barrier to public engagement in CPR. Hung et al.'s [28] 2017 study in Hong Kong revealed that approximately 60 % of surveyed residents would perform CPR on strangers when mouth-to-mouth ventilation was not needed. Similarly, survey findings in Scotland revealed notable reluctance among the local population to perform mouth-to-mouth resuscitation as part of CPR procedures. These findings suggest the need for reevaluation of conventional CPR techniques [67]. The American Heart Association (AHA), which updates its CPR guidelines every five years on the basis of scientific evidence and expert consensus, and revised its recommendations in 2015, and in 2015 revised its recommendations by changing the sequence of conventional CPR to C-A-B, making chest compressions the first step in basic life support and greatly reducing the time of the first cycle [68]. At the same time, untrained first responders are encouraged to perform hands-only CPR [69]. Bobrow et al. [70] observed a substantial increase in bystander CPR rates from 19.6 % in 2005 to 75.9 % following a large-scale public education campaign on hand-only CPR. In another prospective trial involving 336 untrained adults, Bobrow et al. [71] found that participants who viewed a 60-s hands-only CPR training video were more likely to attempt resuscitation and demonstrated better CPR skills than untrained individuals were. However, Karuthan et al.'s [39] research revealed insufficient awareness of hands-only CPR among Malaysian university students. And a recent study conducted in Australia in 2017, only half of those surveyed have heard of hands-only CPR [44]. These findings suggest that enhancing public awareness of hands-only CPR and promoting innovative training methods could significantly improve bystander CPR rates and out-of-hospital cardiac arrest survival rates. This study recommends (1) implementation of innovative training approaches to increase public engagement, (2) the development of standardized, culturally appropriate CPR education programs, and (3) the promotion of hand-only CPR techniques to overcome traditional barriers.

### 5.4 The impact of demographic characteristics and personal factors on bystanders' willingness to perform resuscitation

This study revealed that demographic characteristics and personal factors, influencing gender, age, fear of disease transmission, and prior CPR experience, significantly influence bystanders' willingness to perform CPR. Gender and age emerged as particularly influential factors in determining CPR intervention willingness. These findings present an intriguing gender disparity: while some studies [24,41] have indicated that female bystanders demonstrate greater willingness to perform CPR, contradictory evidence from Pei et al. and the other studies [33,42,49] suggested that male bystanders are more inclined to intervene. This discrepancy warrants further investigation, potentially leading to gender-specific training curricula to enhance public CPR engagement. With respect to age factors, Mao et al. [50] investigation of Chongqing civil servants revealed higher CPR willingness among individuals over 40 years old. Swor et al.'s [30] qualitative interviews revealed greater willingness among bystanders under age 50, and a Korean survey [37] indicated a greater intervention propensity among those under age 60. Collectively, these studies suggest that individuals aged 40–60 years demonstrate the highest CPR willingness, potentially because physical limitations affect older populations. These findings suggest several practical implications for future CPR training programs: (1) development of age-specific training modules, (2) increased focus on elderly and adolescent populations, (3) consideration of physical capability assessments in training design, and (4) implementation of adaptive training methods for different age groups.

Individuals with prior hands-on CPR experience demonstrate superior mastery of resuscitation knowledge and skills. Empirical evidence suggests that most rescuers perceive CPR administration as a positive experience and express willingness to intervene in future cardiac arrest situations [72]. This finding is corroborated by a Swedish study, which revealed that respondents with previous experience in performing bystander CPR during emergency situations exhibited significantly greater willingness to engage in subsequent resuscitation attempts [73].

Concerns about disease transmission during CPR administration constitute another significant barrier to bystander intervention rates. In the United States, 82 % of the general population expresses at least moderate concern about disease transmission [65], whereas in Sweden [27], over 90 % of lay rescuers share similar apprehensions. In contrast, Japanese studies revealed that only 5 % of teachers and high school students perceived disease transmission as a CPR barrier [74], with 18 % of the general public expressing concerns, in Queensland [35]. These geographical disparities may be partially attributable to varying prevalence rates of infectious diseases across these nations.

### 5.5 The impact of legal considerations on bystander resuscitation willingness

The literature review revealed that potential legal litigation and concerns about causing secondary injuries constitute significant barriers to CPR implementation. Public surveys in China, the United Kingdom, and Italy reveal substantial apprehension about potential lawsuits following CPR administration [23,36,51], which may deter bystanders from intervening during cardiac arrest emergencies. Empirical evidence suggests that implementing legal protection for lay rescuers could reduce their ability to perform CPR on strangers by up to 90 % [46]. Recognizing this, numerous countries, including the United States, South Korea, and Canada, have enacted Good Samaritan laws to provide legal protection for bystanders, thereby increasing public willingness to perform CPR.

### 5.6 The effect of research methodologies on public CPR implementation

Self-made questionnaires were used in all studies, which resulted in a variety of research findings and further impacted the conclusions' comparability and validity. To guarantee the consistency and scientific validity of the research findings, future studies can create a standardized evaluation instrument and analyze its validity and reliability. The instrument is specifically used to investigate the influencing factors of public CPR implementation.

### 5.7 Limitations

This study has several limitations that should be acknowledged First, potential data loss may occur during the process of converting quantitative research data using NVivo software, which could affect the accuracy and completeness of the analysis. Second, the relatively limited number of high-quality studies included in our systematic review may constrain the generalizability and robustness of the findings. Finally, the search results and identified factors may have limited applicability across different global regions, as cultural, socioeconomic, and health care system variations may significantly influence the outcomes of public cardiopulmonary resuscitation initiatives.

## 6 Conclusions

Through a rigorous mixed-methods systematic review of multiple databases, this study explored the factors influencing public participation in the OCPR. The investigation revealed numerous determinants affecting bystanders' willingness to resuscitate. The following evidence-based recommendations are proposed: First, future CPR training programs should be designed with consideration of personal factors such as gender and age. The incorporation of female manikins can help alleviate privacy concerns, whereas the use of plain language ensures better comprehension. Training content should include ethical education to promote helping behavior toward strangers. Second, CPR instruction can be mandatory in primary, junior high, and other school curricula in nations such as China which have experienced a slow development of CPR training growth. This will essentially address the issue of low CPR understanding. Third, for community-based training involves the implementation of frequent training sessions and refresher courses to maintain and enhance public CPR competency. Fourth, the establishment of legal protection for lay rescuers through national legislation is crucial for increasing public CPR implementation rates. Finally, to improve the consistency and scientific nature of study findings, researchers should create evaluation instruments in the future that investigate the impact on public cardiopulmonary resuscitation. These comprehensive recommendations address the multifaceted barriers to BCPR and provide a framework for improving OHCA outcomes through educational, practical, legal interventions, and designing specialized evaluation instruments.

## CRediT authorship contribution statement

**Yajie Wang:** Writing – original draft, Methodology, Data curation. **Fangqiu Zheng:** Writing – review & editing. **Jingju Xia:** Methodology, Formal analysis, Data curation. **Limin Zheng:** Methodology, Data curation. **Dantong Wang:** Methodology, Formal analysis. **Haili Wang:** Methodology, Formal analysis. **Bo Ma:** Writing – review & editing, Methodology.

## Funding sources

This mixed-methods review was funded by the fund for projects in Liaoning Province(No.2023JH4/10600033).

## Declaration of competing interest

The authors declare that they have no known competing financial interests or personal relationships that could have appeared to influence the work reported in this paper.
