# Supplementary material for: Influencing factors of public willingness to implement cardiopulmonary resuscitation: A mixed-methods systematic review
Source: Int J Cardiol Cardiovasc Risk Prev. 2025 Sep 24;27:200519. doi: 10.1016/j.ijcrp.2025.200519 (PMC12510220; doi:10.1016/j.ijcrp.2025.200519)
Supplement: Multimedia component 1 [file mmc1.doc]

Supplemental material-1

Using the principle of combining free-text words and subject headings, we searched the four databases of Pubmed, Cochrane library, WOS and Embase respectively.The search period was from had created databases to November 13, 2024.The search is as follows:

Pubmed：

#1 "Cardiopulmonary Resuscitation"[Mesh]

#2((((('Mouth-to-mouth resuscitatio'[Title/Abstract]) OR ('cardio-pulmonary resuscitation'[Title/Abstract])) OR ('Basic cardiac life support'[Title/Abstract])) OR (Resuscitation[Title/Abstract])) OR (CPR[Title/Abstract])) OR ('Code Blue'[Title/Abstract])

(("Cardiopulmonary Resuscitation"[Mesh]) OR ((((((("Mouth-to-mouth resuscitation"[Title/Abstract]) OR ("cardio-pulmonary resuscitation"[Title/Abstract])) OR ("Basic cardiac life support"[Title/Abstract])) OR (Resuscitation[Title/Abstract])) OR (CPR[Title/Abstract])) OR ("Code Blue"[Title/Abstract])) OR ("Cardiopulmonary Resuscitation"[Title/Abstract]))) AND (((Willingness[Title/Abstract]) OR (Behavior[Title/Abstract])) OR (intention[Title/Abstract]))

# 3 #1 or #2

#4 ((Willingness[Title/Abstract]) OR (Behavior[Title/Abstract])) OR (intention[Title/Abstract])

#5 #3 and #4

Cochrane library:

#1 (Cardiopulmonary Resuscitation):ti,ab,kw OR (Cardiopulmonary Resuscitation):ti,ab,kw OR (cardio-pulmonary resuscitation):ti,ab,kw OR (Basic cardiac life support):ti,ab,kw OR (Resuscitation or CPR or Code Blue):ti,ab,kw

#2 (Willingness):ti,ab,kw OR (Behavior):ti,ab,kw OR (intention):ti,ab,kw

#3 #1 and #2

Embase:

#1 'resuscitation'/exp

#2 'bystander cpr':ti,ab,kw OR 'bystander-initiated cpr':ti,ab,kw OR 'cardio pulmonary resuscitation':ti,ab,kw OR 'cardiopulmonary resuscitation':ti,ab,kw OR 'chest compression':ti,ab,kw OR 'reanimation':ti,ab,kw OR 'resuscitation orders':ti,ab,kw OR 'resuscitation':ti,ab,kw

#3 #1 OR #2

#4 willingness OR behavior OR intention

#5 #3 AND #4

WOS：

#1 (((((((TS=("cardiopulmonary resuscitation")) OR TS=("Mouth-to-mouth resuscitation")) OR TS=("cardio-pulmonary resuscitation")) OR TS=("Basic cardiac life support")) OR TS=(Resuscitation)) OR TS=(CPR)) OR TS=("Code Blue")) OR TS=("Cardiopulmonary Resuscitation")

#2 ((TS=(Willingness)) OR TS=(Behavior)) OR TS=(intention)

#3 #1 and 2

Supplemental material-2

supplementary materials

Table 1 Critical Appraisal Skills Programme (CASP) Qualitative Checklist

|  | 1 | 2 | 3 | 4 | 5 | 6 | 7 | 8 | 9 | 10 | Score (%) ^a^ |
| --- | --- | --- | --- | --- | --- | --- | --- | --- | --- | --- | --- |
|  | Clear aims | Suitable qualitative methodology | Appropriate research design | Recruitment | Data collection | Researcher participant relationship | Ethics | Data analysis | Clear statement of findings | Value of research |
| Charlton, K.et al.,2023 | ✓ | ✓ | ✓ | ✓ | ✓ | ? | ✓ | ✓ | ✓ | ✓ | 90 |
| Dobbie, F.et al.,2018 | ✓ | ✓ | ✓ | ✓ | ? | ? | ✓ | ✓ | ✓ | ✓ | 80 |
| Swor, R.et al.,2006 | ✓ | ✓ | ✓ | × | ✓ | ? | ✓ | ✓ | ✓ | ✓ | 80 |
| Son, J. W.et al.,2017 | ✓ | ✓ | ✓ | ✓ | ? | ? | ? | ✓ | ✓ | ✓ | 70 |

✓, High quality for this domain; ×, Low quality for this domain; ?, Unclear quality / insufficient information for this domain. ^a^ Overall score calculated as the percentage of domains for which the study is rated as high quality.

Table 2 Centre for Evidence-Based Management (CEBMa) critical appraisal of a survey tool

| Question number | 1 | 2 | 3 | 4 | 5 | 6 | 7 | 8 | 9 | 10 | 11 | 12 | Score (%) ^a^ |
| --- | --- | --- | --- | --- | --- | --- | --- | --- | --- | --- | --- | --- | --- |
| Domain | Clear research question | Appropriate study design | Clear sample selection methods | Selection bias | Representative sample | Power calculations | Response rate | Valid and reliable questionnaires | Statistical significance assessed | Confidence intervals provided | Confounding | Applicability of results |
| Lu, C.et al.,2016 | ✓ | ✓ | ✓ | ✓ | × | ✓ | ✓ | ✓ | ✓ | ? | ✓ | ✓ | 83 |
| Axelsson, A.et al.,2000 | ✓ | ✓ | ✓ | × | ✓ | ? | ✓ | × | ✓ | ? | ✓ | ✓ | 66 |
| Hung, M. S. Y.et al.,2017 | ✓ | ✓ | ✓ | × | ✓ | ? | ✓ | ✓ | ✓ | ? | × | ✓ | 66 |
| Shams, A.et al.,2016 | ✓ | ✓ | × | × | ✓ | ✓ | ✓ | ? | ✓ | ? | ✓ | ✓ | 66 |
| Urban, J.et al.,2013 | ✓ | ✓ | × | ✓ | × | ✓ | × | × | ✓ | ✓ | ✓ | ✓ | 66 |
| Al-Riyami, H.et al.,2020 | ✓ | ✓ | × | ? | × | ? | ✓ | ✓ | ✓ | ? | × | ✓ | 49 |
| Lu, C.; Jin, Y. H.et al.,2017 | ✓ | ✓ | × | × | ✓ | ✓ | × | ✓ | × | ? | ✓ | ✓ | 58 |
| Chen, Y.et al.,2024 | ✓ | ✓ | × | ✓ | × | ✓ | ✓ | ✓ | ✓ | ? | ✓ | ✓ | 74 |
| Johnston, T. C.et al.,2003 | ✓ | ✓ | × | × | ✓ | ? | ✓ | ? | ✓ | ? | × | ✓ | 50 |
| Riccò, M.et al.,2020 | ✓ | ✓ | × | × | ✓ | ? | ✓ | ✓ | ✓ | ✓ | ✓ | ✓ | 75 |
| Lee, M. J.et al.,2013 | ✓ | ✓ | ✓ | × | ✓ | ? | ✓ | × | ✓ | ✓ | ✓ | ✓ | 75 |
| Alwidyan, M. T.et al.,2023 | ✓ | ✓ | ✓ | × | ✓ | ✓ | × | ✓ | ✓ | ? | ✓ | ✓ | 75 |
| Karuthan, S. R.et al.,2019 | ✓ | ✓ | × | ✓ | × | ✓ | ? | × | ✓ | ? | ✓ | ✓ | 58 |
| Chew, K. S.et al.,2019 | ✓ | ✓ | × | ✓ | × | ? | ? | ✓ | ✓ | ? | ✓ | ✓ | 58 |
| Mao, J.et al.,2021 | ✓ | ✓ | × | × | ✓ | ? | ✓ | ✓ | ✓ | ✓ | ✓ | ✓ | 75 |
| Coons, S. J.et al.,2009 | ✓ | ✓ | ✓ | × | ✓ | ✓ | × | ✓ | ✓ | ✓ | ✓ | ✓ | 83 |
| Charlton, K.et al.,2022 | ✓ | ✓ | ? | ✓ | × | ? | ✓ | × | ✓ | ✓ | ✓ | ✓ | 66 |
| Bray, J. E.et al.,2017 | ✓ | ✓ | ? | × | ✓ | ? | × | ✓ | ✓ | ? | ✓ | ✓ | 58 |
| Anto-Ocrah, M.et al.,2020 | ✓ | ✓ | ✓ | ✓ | × | ✓ | × | × | ✓ | ? | ✓ | ✓ | 66 |
| Chen, M.et al.,2017 | ✓ | ✓ | × | × | ✓ | ? | × | × | ✓ | ? | ✓ | ✓ | 50 |
| Pei-Chuan, H. E.et al.,2019 | ✓ | ✓ | ✓ | × | ✓ | ? | × | ✓ | ✓ | ✓ | ✓ | ✓ | 75 |
| Kuramoto, N.et al.,2008 | ✓ | ✓ | × | × | ✓ | ? | × | × | ✓ | ✓ | ✓ | ✓ | 58 |
| Gul, S.et al.,2019 | ✓ | ✓ | ✓ | ✓ | × | ✓ | ✓ | ✓ | ✓ | × | ✓ | ✓ | 83 |
| Sipsma, K.et al.,2011 | ✓ | ✓ | ? | × | ✓ | ? | × | × | ✓ | × | ✓ | ✓ | 50 |

✓, High quality for this domain; ×, Low quality for this domain; ?, Unclear quality / insufficient information for this domain. ^a^ Overall score calculated as the percentage of domains for which the study is rated as high quality.
